# Comparison of stool collection and storage on Whatman FTA Elute cards versus frozen stool for enteropathogen detection using the TaqMan Array Card PCR assay

**DOI:** 10.1371/journal.pone.0202178

**Published:** 2018-08-30

**Authors:** Tahaniyat Lalani, Michele D. Tisdale, Jie Liu, Indrani Mitra, Cliff Philip, Elizabeth Odundo, Faviola Reyes, Mark P. Simons, Jamie A. Fraser, Emma Hutley, Patrick Connor, Brett E. Swierczewski, Eric Houpt, David R. Tribble, Mark S. Riddle

**Affiliations:** 1 Infectious Disease Clinical Research Program, Department of Preventive Medicine and Biostatistics, Uniformed Services University of the Health Sciences, Rockville, MD, United States of America; 2 Henry M Jackson Foundation for the Advancement of Military Medicine, Bethesda, MD, United States of America; 3 Division of Infectious Diseases, Naval Medical Center, Portsmouth, VA, United States of America; 4 University of Virginia, Charlottesville, VA, United States of America; 5 United States Army Medical Research Unit Kenya, Kericho, Kenya; 6 Joint Task Force Bravo, Soto Cano Air Base, Honduras; 7 Naval Medical Research Center, Silver Spring, MD, United States of America; 8 Department of Military Medicine, Royal Centre for Defense Medicine, Birmingham, United Kingdom; 9 Armed Forces Research Institute for the Medical Sciences, Bangkok, Thailand; 10 F. Edward Hébert School of Medicine, Uniformed Services University, Bethesda, MD, United States of America; George Washington University School of Medicine and Health Sciences, UNITED STATES

## Abstract

The use of Polymerase Chain Reaction (PCR) assays for pathogen detection in travelers’ diarrhea (TD) field studies is limited by the on-site processing and storage requirements for fecal specimens. The objectives of this investigation were to i) characterize the pathogen distribution in deployed military personnel with TD using the TaqMan® Array Card PCR (TAC) on frozen stool and diarrheal smears on Whatman FTA Elute cards (FTA cards), and to ii) compare TAC detection of enteropathogen targets using smeared FTA cards and frozen stool, using TAC on frozen stool as the ‘reference standard’. Stool samples, obtained from active duty personnel with acute TD enrolled in a field trial, were smeared onto FTA cards and stored at room temperature. A corresponding aliquot of stool was frozen in a cryovial. FTA cards and frozen stool samples were tested at a central lab, using a customized TAC for detection of TD pathogens. 187 paired frozen stool samples and smeared FTA cards were stored for a median of 712 days (IQR 396–750) before testing. Overall detection rates were 78.6% for frozen stool and 73.2% for FTA cards. Diarrheagenic *Escherichia coli* were the most common bacteria identified. Using the TAC results on frozen stool as the reference, the overall sensitivity and specificity of TAC on FTA cards was 72.9% and 98.0% respectively. TAC on FTA cards demonstrated a decrease in sensitivity with increasing frozen stool quantification cycle (Cq) (90.0% in FTA cards with a corresponding frozen stool Cq < 30, and 72.9% in samples with a corresponding frozen stool Cq < 35). Our findings support the use and further development of FTA cards in combination with a quantitative PCR assay for enteropathogen detection in TD field studies.

## Introduction

Travelers’ diarrhea (TD) is a common problem during overseas travel and military deployment [1, 2]. Ascertaining the pathogen-specific epidemiology of TD is essential for the understanding of disease burden, as well as development of effective preventive and treatment measures. Traditional enteropathogen detection methods such as stool culture, antigen detection and microscopy have important limitations when applied in a field setting [3]. These labor-intensive and equipment-sensitive methods vary in sensitivity and specificity across pathogens, and are reliant on processing and storage capabilities that are seldom available during military deployments and at overseas travel destinations. As a result, epidemiological data is largely based on one-off intensive field laboratory efforts, and diagnostic decision support in the management of individuals and population health is largely absent.

Several PCR based platforms have been developed for high-throughput, rapid detection of enteropathogens from fecal specimens, that demonstrate increased sensitivity when compared with conventional microbiologic methods [3, 4]. Quantitative, real-time PCR (rt-PCR) assays have the added advantage of permitting an estimation of nucleic acid quantity by the quantification cycle (Cq) [5, 6]. In particular, a customized TaqMan® Array Card (TAC, Life Technologies, Carlsbad, CA) panel for diarrheal pathogens permits spatial multiplexing of up to 384 targets [6, 7].

Filter paper based collection of stool in combination with a quantitative PCR assay would have direct applicability towards clinical diagnostics in austere settings. The Whatman FTA Elute card (FTA card, GE Healthcare Life Sciences, Marlborough, MA, USA) is able to lyse cells, bind PCR inhibitors and store nucleic acid at room temperature for prolonged periods, thus facilitating the collection and transportation of fecal samples to testing sites [8]. Limited data are available on the performance characteristics of PCR assays when performed on smears collected on a filter paper based matrix as compared to fresh or frozen stool [9, 10].

The Trial Evaluating Ambulatory Therapy for TD (TrEAT TD) study evaluated the effectiveness of single dose antibiotic therapy for treatment of ambulatory TD in deployed UK and US military personnel [11]. Subjects provided a stool sample prior to therapy, a portion of which was smeared onto a FTA card and another frozen in a cryovial. The objectives of this investigation were to i) characterize the pathogen distribution in subjects with TD using the TAC and to ii) compare TAC detection rates between FTA cards and corresponding frozen stool samples, using frozen stool as the ‘reference standard’.

## Materials and methods

### Study design

The study design and results of the clinical trial have been previously reported [11]. The study was approved by the Uniformed Services University Infectious Disease Institutional Review Board, United Kingdom Ministry of Defence Research Ethics Committee, and the Kenya Medical Research Institute Ethical Review Committee. Written informed consent was obtained from all participants. No minors were enrolled in the study. Briefly, deployed UK and US military personnel with TD, defined as ≥ 3 unformed stools in 24 hours or > 2 stools in 24 hours associated with nausea, vomiting, abdominal cramps or tenesmus, presenting for care at Camp Bastion (Afghanistan), Camp Lemonnier (Djibouti), British Army Training Unit (Kenya), Soto Cano Air Base (Honduras), and in Thailand during a military exercise were enrolled between September 2012 and July 2015. Acute dysentery or febrile diarrhea (ADF) was defined as symptoms meeting criteria for TD with either fever (i.e. temperature > 100.5°F [38.1°C]) and/or visualized gross blood in diarrheal stool, confirmed by a positive hemoccult test. Subjects with TD who did not meet ADF criteria were classified as having acute watery diarrhea (AWD). Subjects were excluded if they received antibiotic therapy in the 72 hours prior to presentation (excluding malaria prophylaxis).

### Specimen handling

Fresh diarrheal stool specimens were processed by on-site laboratories. Using a wooden applicator, a thin smear of diarrheal stool was applied across a FTA card. The card was air dried for 30 minutes, placed in a multi-barrier pouch with a desiccant, sealed, and stored at room temperature. An aliquot of 180–220 mg of stool (or 200μl if liquid stool) was placed in a cryovial and stored at -80°F. The FTA cards were batch shipped at room temperature, and frozen cryovials of stool were shipped on dry ice to the Naval Medical Center, Portsmouth, VA for TAC testing.

### TAC design

TAC is a microfluidic card with 384 probe based, singleplex rt-PCR reactions. A customized TD TAC using previously published assays for detection of 20 common enteropathogens was used (S2 Table) [6, 7]. The TAC assay also included 14 ETEC colonization factor (CF) and enterotoxin targets that are relevant to pathogenesis and vaccine development [12]. Definitions for pathogen detection using multiple targets are also included in the S2 Table.

### Nucleic acid extraction and TAC testing

Frozen stool samples and corresponding FTA cards for each subject were extracted in the same batch. DNA and RNA were extracted from frozen stool samples using the modified QIAamp Fast DNA Stool Mini Kit extraction protocol (Qiagen, Valencia, CA). In brief; InhibitEX lysis buffer was spiked with two extrinsic controls, 10^6^ copies of phocine herpesvirus (PhHV) and 10^7^ MS2 bacteriophage per sample, to monitor extraction and PCR efficiency. Two hundred mg of frozen stool was lysed with the spiked InhibitEX buffer, beaten for 3 minutes with 212–300 μm glass beads (Sigma, St. Louis, MO), incubated at 95°C for 5 mins and centrifuged for 1 min to pellet the stool particles. Six hundred μL of the lysate was extracted and eluted in 200 μL of elution buffer following the manufacturer’s instructions. Smears on FTA cards were extracted as follows: three, 3 mm discs were punched from the sample area on the FTA card and pulse vortexed with 500 μL of sterile water [8]. The discs were then mixed with 100 μL of sterile water and the two extrinsic controls, subjected to bead beating, and then incubated at 95°C for 5 mins. The eluent was stored at -20°C until testing.

Twenty μL of total nucleic acid extract was mixed with 50 μL of AgPath-ID One-Step rt-PCR buffer, 4 μL of the enzyme mix, and nuclease free water to a 100 μL volume. The reaction mix was loaded into the fill port of the TAC platform and underwent a quantitative PCR on ViiA 7 system. Six samples and two extraction blanks (1 stool and 1 FTA card) were tested on each TAC. Cq cutoffs of 35 were applied, based on data described in prior studies [6, 7]. Positive results were considered valid only when the corresponding extraction blank was negative for the relevant target; negative results were considered valid only when the extrinsic controls were positive for the given samples (dataset for manuscript–S3 Table).

### Statistics

Overall detection rates for TAC on frozen stool and FTA cards, pathogen distribution, and prevalence of enterotoxins and CF among ETEC positive samples were estimated using descriptive statistics. Linear correlation between the Cqs for enterotoxin and primary/sole CF (the most abundant CF type measured by Cq value), secondary CF (the second most abundant) etc. was determined using the Pearson’s correlation coefficient. The sensitivity and specificity of the TAC on FTA card was estimated using TAC on frozen stool as the reference standard. Cqs between the FTA card and frozen stool were compared with the Wilcoxon Signed Rank Test and linear correlation between Cqs was measured by Pearson coefficient. The Cohen's kappa statistic was used to determine the diagnostic agreement between the TAC results on frozen stool and FTA cards. All analyses were performed using SAS version 9.4 (SAS Institute, Cary, NC).

## Results

**Fig 1:**
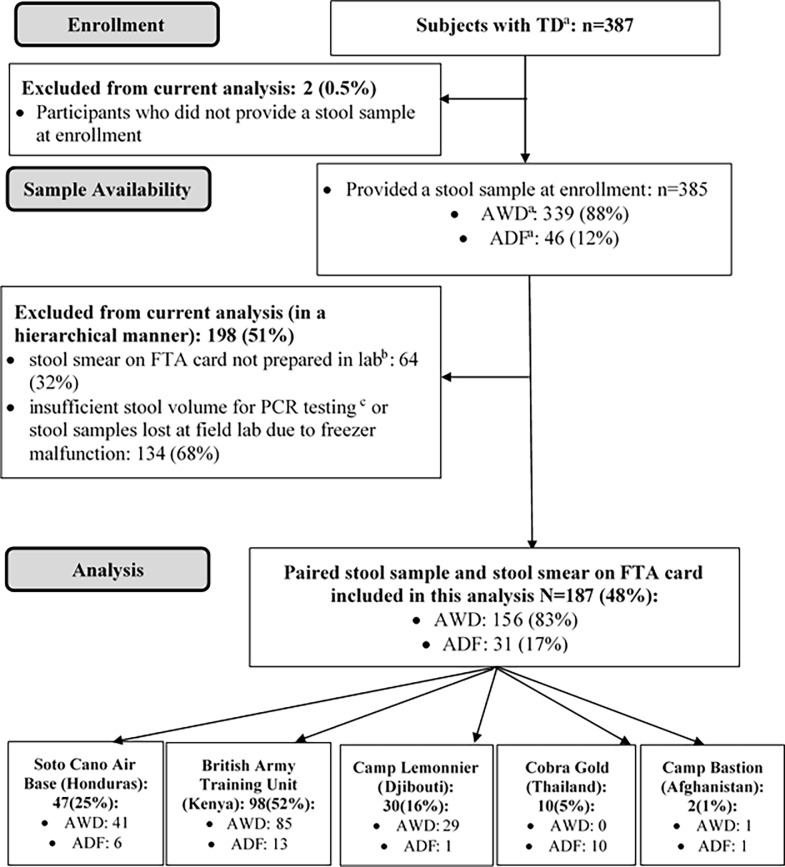
Flow diagram of patients enrolled in the TrEAT TD clinical trial and paired samples that were included in the present analysis.

Three hundred and eighty-seven subjects were enrolled in the study and of these, 187 (48.3%) had paired frozen stool samples and smeared FTA cards available for testing—Fig 1. Frozen stool samples and FTA cards were stored for a median of 712 days (IQR: 396–750 days) before extraction and PCR testing. Extrinsic controls were positive in 98.9% of frozen stool samples and 99.5% of FTA cards (P = NS), with a mean Cq of 28.3 +/- 1.7 vs. 24.6 +/- 1.8, respectively (P<0.01). The average Cq for the bacterial 16S rRNA gene target, an approximate indicator of pathogen burden and starting material for the PCR reaction, was lower for frozen stool samples compared to FTA cards 16.4 +/-3.1 vs. 18.4 +/- 2.2 (P< 0.01). The extrinsic RNA control (MS2) was negative in one paired sample—all RNA negative results for that sample were deemed ‘indeterminate’ and excluded from the analysis.

**Fig 2:**
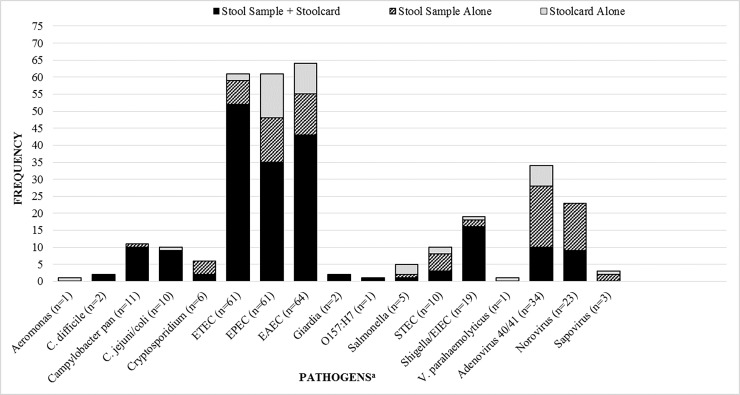
Overall distribution of pathogens in the 187 paired stool and FTA card samples from subjects with travelers’ diarrhea enrolled in the TrEAT TD clinical trial. Footnote for Fig 2. ^a^ Totals in horizontal axis represent total number of subjects positive for a pathogen on either a stool sample, Whatman FTA card or both. No samples were positive for Cyclospora, *Entamoeba histolytica*, astrovirus, and rotavirus and are therefore not shown.

**Fig 3:**
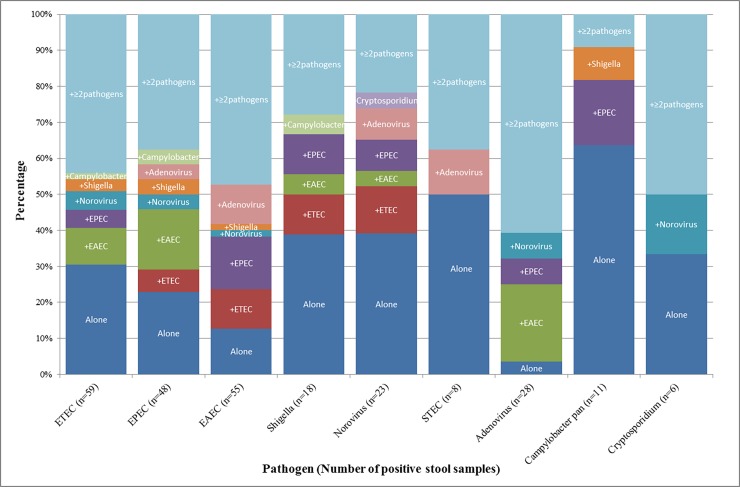
Distribution of solo and co-pathogens detected by the TaqMan Array Card in frozen stool.

Overall pathogen detection rate, distribution and co-pathogen detection: The overall detection rate for enteropathogens using the TAC on frozen stool samples and FTA cards was 78.6% (147/187) and 73.2% (137/187) respectively (p = 0.22), with a combined detection of 82.3% (154/187)—Fig 2. Diarrheagenic *E*. *coli* were the most common bacteria identified and Adenovirus 40/41 and Norovirus were the most common viral pathogens—Fig 2. Among the 31 ADF cases, *C*. *jejuni/coli* was the most common pathogen detected in frozen stool (n = 9), followed by EPEC (n = 8), EAEC (n = 7) and *Shigella*/EIEC spp. (n = 7). Mixed infections were common (frozen stool: 40.6%; average number of pathogens: 1.4 +/- 1.1 pathogens; FTA card: 32.6%; average number of pathogens: 1.2 +/- 1.0 pathogens; p-value for difference in proportions = 0.16). Adenovirus 40/41 was almost always associated with a mixed infection i.e. 96.4% of Adenovirus positive samples had one or more co-pathogens. Mixed infections were also common among known TD pathogens including ETEC (69.5%), EAEC (87.2%) and *Shigella*/EIEC (61.1%) (Fig 3).

Fifty-nine frozen stool samples were ETEC positive: 14 (23.7%) were positive for heat-labile toxin (LT) alone, 26 (44.1%) were positive for heat-stable toxin (ST) alone, and 19 (32.2%) were positive for both LT and ST—Table 1. An additional five samples were LT-ST- but CF+: 2 positive for CS1/PCFO71 alone, 2 positive for CS6 and 1 positive for CS21. At least one CF was present in 83% (49/59) of ETEC positive frozen stool samples, and 38.9% (23/59) were positive for more than one. CS6 was the most common colonization factor detected (61%), and occurred as the sole colonization factor more frequently (38.9%) than others. The Cq of the CF was significantly correlated with the corresponding enterotoxin genes in frozen stool samples (Pearsons coefficient (R): 0.81; (R^2^): 0.66; p<0.01) and FTA card (Pearsons coefficient (R): 0.80; (R^2^): 0.64; p<0.01) (S1 and S2 Figs).

**Table 1:** Distribution of colonization factors among ETEC positive stool samples (n = 59).

Colonization Factor ^a^	ST+ Only (n = 26)	ST/LT+ (n = 19)	LT+ Only (n = 14)
CF negative	3 (11.5)	0	7 (50.0)
CFA/I only	0	0	0
CS1/PCFO71 only	0	0	0
CS2 only	1 (3.8)	0	0
CS3 only	0	1 (5.3)	0
CS5 only	0	0	0
CS6 only	15 (58)	2 (10.5)	6 (42.8)
CS12 only	0	0	0
CS21 only	0	0	1 (7.1)
CFA/I + CS21	4 (15.4)	2 (10.5)	0
CS3 + CS2	0	1 (5.3)	0
CS6 + CS3	1 (3.8)	1 (5.3)	0
CS6 + CS5	1 (3.8)	0	0
CS6 + CS12	0	2 (10.5)	0
CS3 + CS2 + CS21	0	2 (10.5)	0
CS6 + CS2 + CS3	0	1 (5.3)	0
CS6 + CFA/I + CS21	1 (3.8)	1 (5.3)	0
CS3 + CS2 + CS21 + CS12	0	1 (5.3)	0
CS6 + CS1/PCFO71 + CS3 + CS21	0	2 (10.5)	0
CS6 + CS2 + CS3 + CS21	0	1 (5.3)	0
CS6 + CS2 + CS3 + CS21+CS1/PCFO71	0	1 (5.3)	0
CS6 + CS2 + CS3 + CS21 + CFA/I	0	1 (5.3)	0

^a^ No samples were positive for CS4, CS7, CS8, CS14, CS17/19, CS18.

### FTA card vs. frozen stool

Considering all 963 positive targets from either frozen stool or FTA cards, the overall positive percent agreement was 76.1% and the negative percent agreement was 99.3%. When corrected for agreement by chance alone with the kappa statistic, the overall diagnostic agreement (κ) was 0.80 (95% CI: 0.77–0.82). The agreement for bacterial targets (κ = 0.82; 95% CI: 0.80–0.85) was higher than for viral targets (κ = 0.48; 95% CI: 0.34–0.62). Using the TAC results on frozen stool as the reference, the overall pathogen detection sensitivity and specificity for TAC on FTA cards was 72.9% and 98.0% respectively. The sensitivity increased to 90.0% when restricting to FTA cards with a corresponding frozen stool Cq < 30—Table 2. The sensitivity of TAC for detection of 14 ETEC CFs and enterotoxins on FTA cards was also frozen stool Cq dependent (S1 Table). Most bacterial pathogens were associated with a higher sensitivity compared with norovirus and *Cryptosporidium*. FTA cards yielded higher Cqs for most targets versus corresponding frozen stool (average ΔCq across all targets = 3.25 +/- 2.75) Table 2. A moderate or weak positive linear relationship (r) between FTA card and frozen stool Cq values for observed for most pathogens except Adenovirus 40/41. The coefficient of determination (r^2^), defined as the proportion of the variance in frozen stool sample Cq that is predictable from the FTA card Cq, was also low for most pathogens except Adenovirus 40/41.

**Table 2:** Comparison of enteropathogen detection on 187 paired stool samples and stool smears stored on FTA Whatman Elute Cards using the TaqMan Array Card PCR platform. PCR results on stool samples used as reference standard^a^.

Pathogen	Stool positive Cq < 30			Stool positive Cq < 35			Stool negative			Cq correlation (both Cqs < 35)			Average Cq, SD (both Cqs < 35)		
	FTA Card +	FTA Card -	Sensitivity % (95%CI)	FTA Card +	FTA Card -	Sensitivity % (95%CI)	FTA Card + Cq < 30	FTA Card + Cq < 35	Specificity % (95%CI)	r	r^2^	P^b^	FTA Card	Stool	P^c^
**ETEC**	54	0	100.0 (93.4–100.0)	77	8	90.6 (82.2–96.0)	0	3	97.6 (93.3–99.5)	0.49	0.24	**<0.01**	25.8 (4.2)	25.9 (4.8)	0.97
**EPEC**	30	1	96.8 (83.3–99.9)	37	13	74.0 (62.0–86.2)	2	12	90.8 (86.0–96.0)	0.59	0.35	**<0.01**	29.3 (3.2)	27.9 (3.0)	**<0.01**
**EAEC**	42	4	91.3 (79.2–98.0)	56	16	77.8 (68.2–87.4)	3	10	93.0 (88.8–97.0)	0.53	0.28	**<0.01**	28.6 (3.3)	27.2 (3.8)	**<0.01**
**Shigella/EIEC**	16	0	100.0 (79.4–100.0)	16	2	88.9 (65.2–98.6)	0	1	99.4 (96.8–99.9)	0.4	0.16	0.13	25.0 (3.0)	24.0 (3.8)	0.40
**Campylobacter**	6	0	100.0 (54.0–100.0)	10	1	90.9 (58.7–99.8)	0	0	100.0 (98.0–100.0)	0.58	0.33	0.08	23.4 (3.3)	28.8 (4.1)	<0.01
**STEC**	1	1	50.0 (1.2–98.7)	4	5	44.4 (14.0–78.8)	0	2	98.8 (96.0–99.8)	0.48	0.23	0.52	28.2 (3.2)	30.8 (4.0)	0.25
**Norovirus**	9	10	47.4 (24.4–71.1)	9	15	38.0 (18.8–60.0)	0	0	100.0 (98.9–100.0)	0.47	0.22	0.20	31.7 (2.1)	23.9 (2.9)	**<0.01**
**Adenovirus 40/41**	6	0	100.0 (54.0–100.0)	10	18	36.0 (18.6–56.0)	0	6	96.2 (92.0–98.6)	0.98	0.96	**<0.01**	27.8 (6.0)	26.5 (7.8)	0.11
**Cryptosporidium**^d^	2	3	40.0 (14.6–94.7)	2	4	33.3 (22.2–96.0)	0	0	100.0 (98.0–100.0)	-	-	-	28.6 (5.2)	28.5(0.2)	1.00
**Total**^d^	166	19	90.0 (85.4–94.1)	221	82	72.9 (68.0–78.0)	5	34	98.0 (97.2–98.6)	-	-	-	27.3 (4.2)	26.6 (4.4)	**<0.01**

^a^ Pathogens in table are limited to those for whom at least 5 stool samples were positive

^b^ Pearson’s Coefficient for correlation data

^c^ Wilcoxon Signed Ranks Test

^**d**^ Cryptosporidium Cq correlation not calculated due to the small sample size. Cq correlation was not calculated for the overall (total) group due to the variability of r and r^2^ between pathogens which limits the interpretability of the estimate

We were unable to determine the impact of storage duration of FTA cards on TAC sensitivity by pathogen since the majority of cards were stored for over 1 year and no cards were stored for less than 6 months (FTA cards stored 6m-1y (n = 29)–sensitivity: 73.6% (95% CI: 48–90%), 1y-2y (n = 90): 95.8% (95% CI: 88–99%), >2y (n = 68): 81.8% (95% CI: 67–92%)).

## Discussion

Our findings demonstrate the performance characteristics of a quantitative PCR when applied to FTA cards, a more feasible alternative to conventional stool collection in the field setting. The advantages of using FTA cards in resource-limited settings include the limited stool volume needed for testing, ability to store samples at room temperature and ship to laboratories using regular mail, thus avoiding the need for expensive biohazard containers and dry ice [13]. Stool samples for 35% of enrolled subjects were either lost due to a freezer malfunction or had an inadequate volume for TAC testing, but corresponding smeared FTA cards were available for testing–Fig 1. Using the TAC results on frozen stool as the reference, the overall sensitivity and specificity for TAC on FTA cards was 72.9% and 98.0% respectively, despite being stored at room temperature in austere environments for a median of 2 years before extraction and testing.

Since an accurate assessment of pathogen load is critical to the use of the FTA cards and TAC assay in field studies, we compared Cq values across pathogen targets in FTA cards and corresponding frozen stool samples. Cq values were higher in FTA cards compared to frozen stool, and a loss in TAC sensitivity on FTA cards was noted at lower pathogen loads in frozen stool. The lower detection rate for TAC on FTA cards compared with frozen stool is likely related to the difference in sampling volumes (200 μL of frozen stool vs. approximately < 20 μL from punches of the FTA card) as well as the degradation of genomic material with prolonged storage [14, 15]. Cq values for the 16S rRNA target, an indirect measure of pathogen load and starting material for PCR reaction, was lower in frozen stool compared to FTA cards (16.4 +/-3.1 vs. 18.4 +/- 2.2; p< 0.01), roughly corresponding to a 4 fold difference in DNA content. Degradation of genomic material due to prolonged storage was suggested by the high number of false negative FTA cards for Norovirus, an RNA virus prone to degradation with prolonged storage, and the significantly higher Norovirus Cq values in FTA cards compared to frozen stool (31.7 +/-2.1 vs. 23.9 +/- 2.9; p< 0.01). Additional internal validation studies using direct measures of DNA/RNA quantity (i.e. by spectrophotometry) and purity (e.g. ratio of the absorbance at 260nm and 280nm) of FTA card smears are needed to better understand differences in Cq values between FTA cards and frozen stool. We were also unable to directly assess the impact of prolonged storage on the performance characteristics of the TAC on FTA cards, since all samples were stored for > 6 months. However, prior studies have demonstrated good recovery of RNA viruses from FTA cards stored for short durations (1–3 months) at room temperature [10, 15, 16].

The TAC demonstrated good sensitivity and specificity for diarrheagenic *E*. *coli* on FTA cards, especially at high pathogen loads. Discordant results were noted at low level detections (i.e. positive FTA card with Cq between 30–35 and negative frozen stool) which could be related to differences in sampling or greater PCR inhibition in frozen stool vs. FTA cards, as evidenced by the lower Cq values for extrinsic controls in the FTA cards versus frozen stool [14, 15]. The FTA card is impregnated with denaturants and chelating agent designed to trap PCR inhibitors and other debris, which are then removed by the washing reagent. FTA card Cq values accounted for a small proportion of variance in frozen stool Cq values across enteropathogen targets (coefficient of determination (r^2^) range: 0.16 to 0.96), indicating that unmeasured factors such as duration and conditions of storage, sampling technique, target specific factors (e.g. plasmids or viral targets prone to degradation), PCR efficiency etc. may also need to be considered in models using FTA card Cqs to predict pathogen load in frozen stool. Furthermore, the findings demonstrate the need to optimize collection and storage and expediency of testing of fecal smears on FTA cards in order to minimize variability due to environmental and sampling conditions. Determining the optimal amount of FTA card sample area for extraction is another key challenge. This is especially pertinent for quantitative PCR testing of differing types of diarrheal specimens. For example, watery diarrhea samples maybe more dilute and therefore require a larger sample area to be tested. We used a Whatman FTA Elute extraction protocol that was available at the time of testing [8]. Since then, newer protocols have recommended using a larger sample area and specific elution buffers instead of water, but the difference in DNA/RNA extraction between protocols has not been evaluated [17].

A substantial proportion of detections yielded mixed infections. For example, of the 59 ETEC positive frozen stool specimens by TAC, 28.8% (n = 17) were single-pathogen detections -Fig 3. Since this analysis was part of a clinical trial focused on TD treatment, we did not include an asymptomatic comparator arm, an approach that has been used to demonstrate the association between detection of certain pathogen and TD attribution [18]. In a study in UK travelers, cases of ETEC gastroenteritis were associated with a significantly higher pathogen burden (i.e. lower ETEC Cq values) compared to controls (mean Cq: 24 vs. 33) [19]. Of note, the mean Cq value for ETEC positive frozen stool samples in our cohort (25.9) was comparable to the mean Cq in ETEC cases reported in the UK travel cohort, potentially supporting the attribution of diarrhea to ETEC. Although we attempted to examine differences in Cq values between primary and secondary pathogens in cases of mixed infections (data not shown), it was difficult to interpret findings without an asymptomatic comparator arm. However, our results suggest that multi-pathogen exposure in travelers may not be at the levels that are found in children under 5 years of age in lower middle income countries. In a cohort of children < 1 year of age from Bangladesh, the mean number of enteropathogen targets was 5.6 ± 0.1 in diarrheal samples and 4.3 ± 0.1 in asymptomatic surveillance samples. After adjusting for pathogens found in control samples, diarrheal specimens appeared to have ≥ 3 probable enteropathogen contributors to diarrhea. [20] In contrast, a case-control study of adults and pediatric subjects with TD, the mean number of pathogens in diarrheal and asymptomatic samples was 1.3 and 1.13 respectively, similar to the mean number of co-pathogens detected in our cohort. [19] Further research is needed to understand how pathogens in combination may attribute to disease, versus the notion of one or the other being a non-contributing bystander. An important goal of using molecular diagnostics in TD field studies and clinical trial is dependent on the ability to optimize collection and storage methods, minimize variability due to PCR inhibitors, and define thresholds for differentiating true pathogen attributable cases using quantitative and/or semi-quantitative PCR assays.

Among ETEC positive cases, the proportions of LT only (23.7%), ST only (44%), and LT+/ST+ (32%) frozen stool detected by TAC were in line with prior reports using culture based testing [21]. TAC detected ≥1 CF in 83% of ETEC cases, significantly higher than detection rates using culture followed by genotypic/phenotypic methods (66%) [12, 21]. The instability of plasmids during culture and potential sampling error while picking colonies could explain the lower detection rate using standard methods. Our CF detection rate was also higher than the 78% rates reported previously using a similar TAC assay in a childhood diarrhea cohort, potentially due to the inclusion of both diarrheal and non-diarrheal samples in that study [12]. A strong positive correlation between Cqs for enterotoxin and CFs was also observed (S1 and S2 Fig), further validating the use of direct on stool TAC for toxin and CF detection. An important limitation of direct on stool TAC is the inability to distinguish CF profiles between ETEC strains, although our findings suggest this should be taken in context with the significant increase in enterotoxin and CF gene detections using direct on stool TAC. The pattern of CF distribution by TAC in our study followed conventional paradigms in travel cohorts [22, 23]. Among ST only ETEC samples (n = 26), 86% were positive for either CS6 or CS21, and 57% were positive for CS6 as the sole CF. Similar findings were reported in a phase 3 trial of an oral LT ETEC vaccine that was not immunogenic to ST or CS6. 17 of the 28 (60.7%) ETEC associated TD cases in the vaccine arm were ST only or LT and ST positive strains with CS6 as the sole CF (n = 16 and n = 1 respectively) [24]. Understanding the ETEC enterotoxin and CF gene profiles in TD cases and controls will be important for informing CF target selection for future vaccine efforts, in addition to other considerations such as immunogenicity.

This study has some limitations including the small sample size for infrequently occurring pathogens and the inability to evaluate the impact of standardization of quantity, self-collection of stool, presence of blood in stool, environmental conditions, and storage duration on detection using FTA cards, especially for RNA viruses. Due to delays in executing agreements, procurement of TAC and shipping of frozen stool samples from overseas locations, samples were stored for a median of 2 years before testing. Although we did not compare TAC to standard microbiologic methods, this is being planned for a separate analysis and has extensively been done previously (6). As noted above, culture based methods have certain advantages including the ability to determine the genotypic and phenotypic profile of ETEC isolates, and therefore should be incorporated on a subset of specimens when feasible.

Overall, our findings suggest that FTA cards are a promising alternative for stool collection in resource limited settings. Additional studies are needed to optimize sample collection and storage, as well as defining thresholds for pathogen attribution in TD cases, using semi-quantitative PCR assays.

## Supporting information

S1 Fig Correlation of Cqs between enterotoxin and CFs on stool (N = 49). **Each symbol represents one CF type. The Cq correlation between enterotoxin and primary/sole CF (the most abundant CF type measured by Cq values), secondary CF (the second most abundant, if present), tertiary CF (the third most abundant, if present), quaternary CF (the fourth abundant, if present).** Correlation coefficient, coefficient of determination and p-value: i) overall (n = 49): R:0.81; R2:0.66; p < 0.001; ii) primary/sole CF (n = 49):R = 0.85, R2 = 0.73, p<0.01; iii) secondary CF (n = 23): R = 0.72, R^2^ = 0.53, p<0.01; iv) tertiary CF (n = 11): R = 0.99, R2 = 0.97, p<0.01; v) quaternary CF (n = 6): R = 0.88, R^2^ = 0.77, p = 0.22.
(TIF)

S2 Fig Correlation of Cqs between enterotoxin and CFs on FTA card (N = 43). **Each symbol represents one CF type. The Cq correlation between enterotoxin and primary/sole CF (the most abundant CF type measured by Cq values), secondary CF (the second most abundant, if present), tertiary CF (the third most abundant, if present), quaternary CF (the fourth abundant, if present).** Correlation coefficient, coefficient of determination and p-value: i) overall (n = 43): R:0.80; R^2^:0.64; p<0.01; ii) primary/sole CF (n = 43):R = 0.86, R^2^ = 0.75, p<0.01; iii) secondary CF (n = 19): R = 0.53, R^2^ = 0.28, p<0.02; iv) tertiary CF (n = 10): R = 0.95, R^2^ = 0.91, p<0.01; v) quaternary CF (n = 5): R = -0.46, R^2^ = 0.21, p = 0.44.
(TIF)

S1 Table Comparison of enterotoxin and CF detection on 59 ETEC positive stool samples and corresponding FTA cards using the TaqMan® Array Card PCR platform. PCR results on stool samples used as reference standard.
(DOCX)

S2 Table Pathogens targeted in this study by the TaqMan Array Card PCR Assaya. ^a^Pathogens with multiple gene targets–definitions for positive detection:
-ETEC: detection of LT, STh or STp
LT only ETEC: LT detected without STh or STpST only ETEC: STh or STp detected without LTLT+/ST+ ETEC: LT detected with either STh or STp-EAEC: detection of either aaiC or aatA-EPEC: detection of either typical or atypical EPEC
Typical EPEC (tEPEC): detection of both eae and bfpAAtypical EPEC (aEPEC): detection of eae without bfpA, stx1, and stx2-STEC: detection of stx1 or stx2-*C*. *difficile*: detection of tcdA or tcdB-Norovirus: detection of Norovirus I or Norovirus II target.
(DOCX)

S3 Table Dataset for manuscript. (XLSX)
