# Supplementary figures and images for: Comparison of stool collection and storage on Whatman FTA Elute cards versus frozen stool for enteropathogen detection using the TaqMan Array Card PCR assay
Source: PLoS One. 2018 Aug 30;13(8):e0202178. doi: 10.1371/journal.pone.0202178 (PMC6117160; doi:10.1371/journal.pone.0202178)

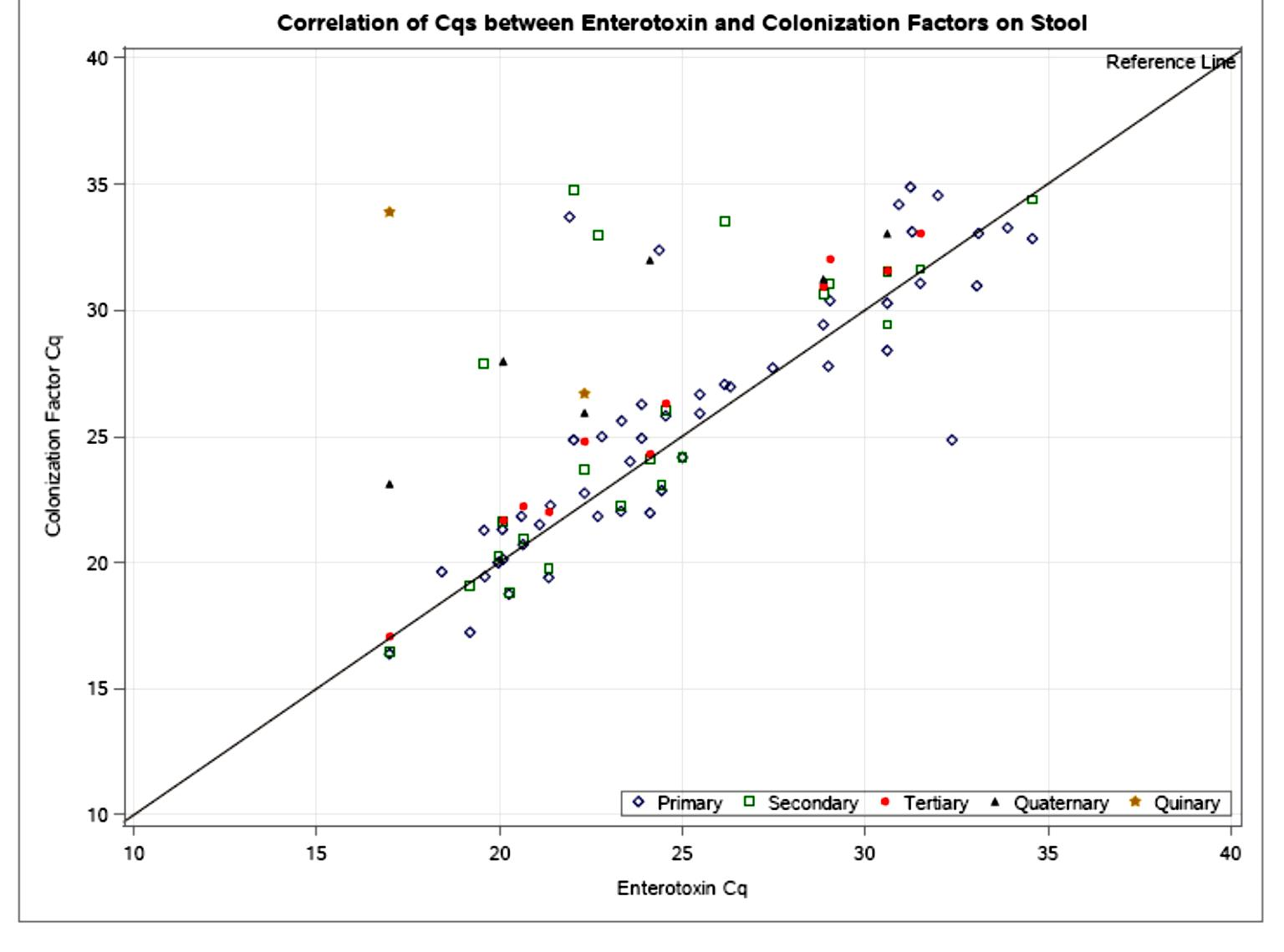

Supplement: S1 Fig — Each symbol represents one CF type. The Cq correlation between enterotoxin and primary/sole CF (the most abundant CF type measured by Cq values), secondary CF (the second most abundant, if present), tertiary CF (the third most abundant, if present), quaternary CF (the fourth abundant, if present). Correlation coefficient, coefficient of determination and p-value: i) overall (n = 49): R:0.81; R2:0.66; p < 0.001; ii) primary/sole CF (n = 49):R = 0.85, R2 = 0.73, p<0.01; iii) secondary CF (n = 23): R = 0.72, R2 = 0.53, p<0.01; iv) tertiary CF (n = 11): R = 0.99, R2 = 0.97, p<0.01; v) quaternary CF (n = 6): R = 0.88, R2 = 0.77, p = 0.22. (TIF) [file pone.0202178.s001.tif]

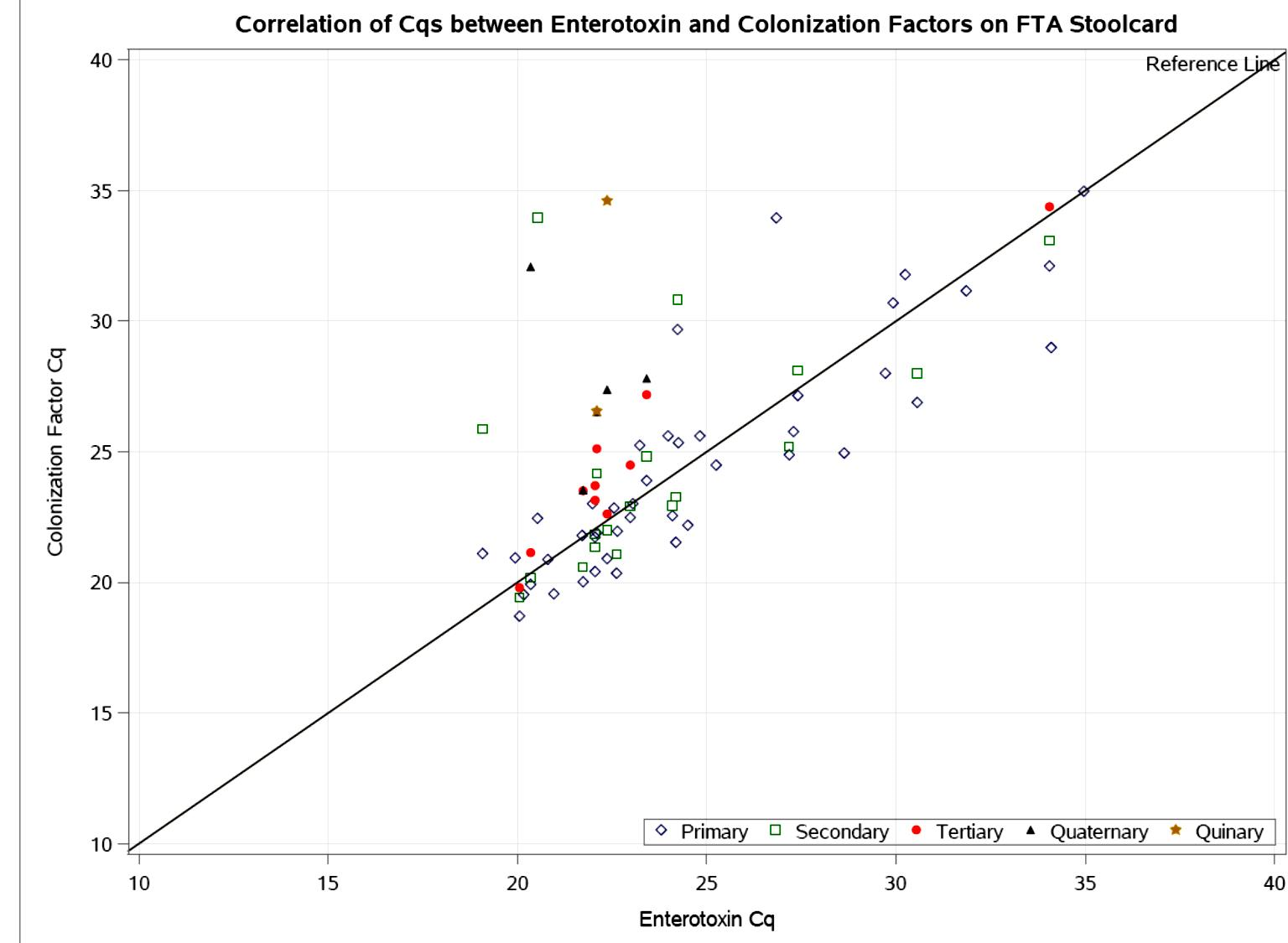

Supplement: S2 Fig — Each symbol represents one CF type. The Cq correlation between enterotoxin and primary/sole CF (the most abundant CF type measured by Cq values), secondary CF (the second most abundant, if present), tertiary CF (the third most abundant, if present), quaternary CF (the fourth abundant, if present). Correlation coefficient, coefficient of determination and p-value: i) overall (n = 43): R:0.80; R2:0.64; p<0.01; ii) primary/sole CF (n = 43):R = 0.86, R2 = 0.75, p<0.01; iii) secondary CF (n = 19): R = 0.53, R2 = 0.28, p<0.02; iv) tertiary CF (n = 10): R = 0.95, R2 = 0.91, p<0.01; v) quaternary CF (n = 5): R = -0.46, R2 = 0.21, p = 0.44. (TIF) [file pone.0202178.s002.tif]
