# Supplementary material for: Comparison of stool collection and storage on Whatman FTA Elute cards versus frozen stool for enteropathogen detection using the TaqMan Array Card PCR assay
Source: PLoS One. 2018 Aug 30;13(8):e0202178. doi: 10.1371/journal.pone.0202178 (PMC6117160; doi:10.1371/journal.pone.0202178)
Supplement: S1 Table — PCR results on stool samples used as reference standard. (DOCX) [file pone.0202178.s003.docx]

**S1 Table.** Comparison of enterotoxin and CF detection on 59 ETEC positive stool samples and corresponding FTA cards using the TaqMan® Array Card PCR platform. PCR results on stool samples used as reference standard

|  | **Stool positive Cq < 30** | | | **Stool positive Cq < 35** | | | **Stool negative** | | | **Cq correlation (both Cqs < 35)** | | **Average Cq, SD (both Cqs < 35)** | | |
| --- | --- | --- | --- | --- | --- | --- | --- | --- | --- | --- | --- | --- | --- | --- |
|  | **FTA Card +** | **FTA Card -** | **Sensitivity %** | **FTA Card +** | **FTA Card -** | **Sensitivity %** | **FTA Card + Cq < 30** | **FTA Card + Cq < 35** | **Specificity %** | **R^2^** | **P-value** | **FTA Card** | **Stool** | **P-value** |
| **LT+ only** | 5 | 0 | 100.0  (48.0-100.0) | 14 | 2 | 87.5  (61.6-98.4) | 0 | 0 | 100.0  (94.1-100.0) | 0.33 | 0.02 | 31.0(5.1) | 30.2(3.0) | 0.18 |
| **ST+ only** | 21 | 0 | 100.0  (84.0-100.0 | 23 | 4 | 85.2  (66.2-96.0) | 0 | 2 | 75.0 (35.0-96.8) | 0.57 | <0.001 | 27.0(6.5) | 25.4(4.4) | 0.05 |
| **ST+LT+** | 28 | 0 | 100.0  (88.0-100.0) | 40 | 2 | 95.2  (84.0-99.4) | 0 | 0 | 100.0  (94.1-100.0) | 0.23 | 0.001 | 25.6(4.8) | 26.0(5.6) | 0.67 |
| **CFA/I** | 6 | 2 | 75.0  (35.0-96.8) | 6 | 4 | 60.0  (26.2-88.0) | 0 | 1 | 98.2  (90.4-99.9) | 0.55 | 0.1 | 25.4(4.0) | 25.4(6.0) | 0.06 |
| **CS1/PCFO71** | 3 | 0 | 100.0  (29.2-100.0) | 4 | 1 | 80.0  (28.4-99.5) | 0 | 0 | 100.0  (94.1-100.0) | 0.41 | 0.36 | 20.8(2.0) | 26.6(5.7) | 0.25 |
| **CS2** | 5 | 0 | 100.0  (48.0-100.0) | 7 | 2 | 77.8  (40.0-97.2) | 0 | 0 | 100.0  (94.0-100.0) | 0.19 | 0.32 | 24.8(2.6) | 26.2(6.3) | 0.02 |
| **CS3** | 7 | 0 | 100.0  (59.0-100.0) | 11 | 2 | 84.6  (54.5-98.0) | 0 | 0 | 100.0  (93.2-100.0) | 0.15 | 0.24 | 23.7(3.1) | 26.3(6.0) | <0.01 |
| **CS6** | 24 | 1 | 96.0  (79.6-99.9) | 31 | 8 | 79.5  (63.5-90.7) | 0 | 2 | 92.6  (76.0-99.0) | 0.13 | 0.05 | 25.6(4.6) | 27.2(4.8) | 0.32 |
| **CS12** | 2 | 0 | 100.0  (16.0-100.0) | 3 | 0 | 100.0  (29.2-100.0) | 0 | 0 | 100.0  (94.3-100.0) | 0.98 | 0.11 | 29.1(4.3) | 29.6(2.9) | 0.25 |
| **CS21** | 11 | 2 | 84.6  (54.6-98.0) | 14 | 4 | 77.8  (52.4-94.0) | 0 | 0 | 100.0  (92.6-100.0) | 0.34 | 0.03 | 22.9(3.6) | 25.0(5.7) | 0.85 |
| **CS5** | 1 | 0 | 100.0  (2.5-100.0) | 1 | 0 | 100.0  (2.5-100.0) | 0 | 0 | 100.0  (94.4-100.0) | N/A | N/A | 22.6(-) | 24.2(-) | 1.00 |
| **Total** | 113 | 5 | 94.8  (89.2-98.1) | 154 | 29 | 80.6  (74.0-86.0) | 0 | 5 | 98.2  (96.4-99.2) | - | - | 24.8(3.8) | 25.1(4.6) | 0.90 |
