# Supplementary material for: Comparison of stool collection and storage on Whatman FTA Elute cards versus frozen stool for enteropathogen detection using the TaqMan Array Card PCR assay
Source: PLoS One. 2018 Aug 30;13(8):e0202178. doi: 10.1371/journal.pone.0202178 (PMC6117160; doi:10.1371/journal.pone.0202178)
Supplement: S2 Table — aPathogens with multiple gene targets–definitions for positive detection: -ETEC: detection of LT, STh or STp LT only ETEC: LT detected without STh or STpST only ETEC: STh or STp detected without LTLT+/ST+ ETEC: LT detected with either STh or STp-EAEC: detection of either aaiC or aatA-EPEC: detection of either typical or atypical EPEC Typical EPEC (tEPEC): detection of both eae and bfpAAtypical EPEC (aEPEC): detection of eae without bfpA, stx1, and stx2-STEC: detection of stx1 or stx2-C. difficile: detection of tcdA or tcdB-Norovirus: detection of Norovirus I or Norovirus II target. (DOCX) [file pone.0202178.s004.docx]

**S2 Table.** Pathogens targeted in this study by the TaqMan Array Card PCR Assay

|  | **Organism** | **Target** | **Description** | **Ref** |
| --- | --- | --- | --- | --- |
| 1. | Astrovirus | *Capsid* | Capsid protein | 6 |
| 2. | Rotavirus | NSP3 | Non-structural protein - shuts off host cell protein synthesis | 6 |
| 3. | Norovirus GI | ORF1-2 | Non-structural polyprotein and capsid protein | 12 |
| 4. | Norovirus GII | ORF1-2 | Non-structural polyprotein and capsid protein | 6 |
| 5. | Adenovirus 40/41 | Hexon | Major coat protein | 6 |
| 6. | Sapovirus | RdRp | RNA dependent RNA polymerase | 6 |
| 7. | Aeromonas | Aerolysin | channel-forming toxin | 12 |
| 8. | *Campylobacter jejuni/coli* | cadF | Outer membrane protein | 6 |
| 9. | Campylobacter pan | cpn60 | chaperonin 60 | 12 |
| 10. | Salmonella spp. | invA | Invasive gene A | 6 |
| 11. | *V. parahaemolyticus* | toxR | transcriptional activator | 12 |
| 12. | Shigella/EIEC | ipaH | Invasion plasmid antigen H | 6 |
| 13. | EPEC | eae | Intimin | 6 |
| 14. |  | bfpA | Bundle forming pilus | 6 |
| 15. | STEC O157:H7 | rfbE | encoding CDP-tyvelose epimerase | 12 |
| 16. | STEC | stx1 | Shiga toxin 1 | 6 |
| 17. |  | stx2 | Shiga toxin 2 | 6 |
| 18. | EAEC | aaiC | Secreted protein | 6 |
| 19. |  | aatA | Dispersin transporter protein | 6 |
| 20. |  | aggR | Transcriptional activator | 12 |
| 21. | ETEC | STh | Heat stable | 18 |
| 22. |  | STp | Heat stable | 18 |
| 23. |  | LT | Heat-labile | 18 |
| 24. |  | CFA/I (cfaB) | Colonization factor antigen | 18 |
| 25. |  | CS1/PCF071 (csoA and cosA) | Colonization factor antigen | 18 |
| 26. |  | CS2 (cotA) | Colonization factor antigen | 18 |
| 27. |  | CS3 (cstA) | Colonization factor antigen | 18 |
| 28. |  | CS4 (csaB) | Colonization factor antigen | 18 |
| 29. |  | CS5 (csfA) | Colonization factor antigen | 18 |
| 30. |  | CS6 (cssB) | Colonization factor antigen | 18 |
| 31. |  | CS7 (csvA) | Colonization factor antigen | 18 |
| 32. |  | CS8 (cofA) | Colonization factor antigen | 18 |
| 33. |  | CS12 (cswA) | Colonization factor antigen | 18 |
| 34. |  | CS14 (csuA) | Colonization factor antigen | 18 |
| 35. |  | CS18 (fotA) | Colonization factor antigen | 18 |
| 36. |  | CS17/19 (csbA and csdA) | Colonization factor antigen | 18 |
| 37. |  | CS21 (lngA) | Colonization factor antigen | 18 |
| 38. | *Clostridium difficile* | tcdA | Protein enterotoxin | 6 |
| 39. |  | tcdB | Protein enterotoxin | 6 |
| 40. | Cyclospora | 18S | Ribosomal RNA | 12 |
| 41. | *Entamoeba histolytica* | 18S | Ribosomal RNA | 6 |
| 42. | Cryptosporidium | 18S | Ribosomal RNA | 6 |
| 43. | Giardia | 18S | Ribosomal RNA | 6 |

**Pathogens with multiple gene targets – definitions for positive detection:**

- ETEC: detection of LT, STh or STp

- LT only ETEC: LT detected without STh or STp
- ST only ETEC: STh or STp detected without LT
- LT+/ST+ ETEC: LT detected with either STh or STp

- EAEC: detection of either aaiC or aatA

- EPEC: detection of either typical or atypical EPEC

- Typical EPEC (tEPEC): detection of both eae and bfpA
- Atypical EPEC (aEPEC): detection of eae without bfpA, stx1, and stx2

- STEC: detection of stx1 or stx2

- *C. difficile*: detection of tcdA or tcdB

- Norovirus: detection of Norovirus I or Norovirus II target
